# Validation and normative data for the BAC app in Spanish-speaking individuals with psychosis and healthy controls

**DOI:** 10.1192/j.eurpsy.2025.10076

**Published:** 2025-08-11

**Authors:** César González-Blanch, Esther Pousa, Pablo Reguera-Pozuelo, Alfonso Gutiérrez-Zotes, Roberto Rodriguez-Jimenez, Manuel del-Castillo-Serrano, Ana González-Pinto, Guillermo Cano-Escalera, Marta Zubia, Joan Vicent Sánchez-Ortí, Patricia Correa-Ghisays, Vicent Balanzá-Martínez, Raquel López-Carrilero, Teresa Bobes-Bascarán, Ana Catalán, Claudia Aymerich, Olimpia Díaz-Mandado, Daniel Guinart, Elisabet Vilella, Leticia García-Álvarez, Susana Ochoa, Salvador Miret, Cristina Falip, Rafael Fernández-Martínez, Ignacio García-Cabeza, Carlos Campos-Rodriguez, Josep Maria Crosas, Jesus Cobo, Helena Pardina-Torner, Salvador Perona-Garcelán, Ángel Yorca-Ruiz, Víctor Ortiz-García de la Foz, Rosa Ayesa-Arriola

**Affiliations:** 1 Mental Health Centre, University Hospital Marqués de Valdecilla, Santander, Spain; 2 IDIVAL, Valdecilla Biomedical Research Institute, Santander, Spain; 3 Department of Psychology, International University of La Rioja (UNIR), Logroño, Spain; 4 Department of Psychiatry, Hospital de la Santa Creu i Sant Pau, Institut de Recerca Sant Pau (IR SANT PAU), Barcelona, Spain; 5 Centro de Investigación Biomédica en Red de Salud Mental (CIBERSAM), Instituto de Salud Carlos III (ISCIII), Madrid, Spain; 6 Department of Psychiatry, School of Medicine, University of Seville, Seville, Spain; 7 Translational Psychiatry Group, Seville Biomedical Research Institute (IBiS)-CSIC, Seville, Spain; 8 CIBERSAM-ISCIII (Biomedical Research Networking Centre in Mental Health), Spain; 9 Department of Psychiatry, Hospital Universitari Institut Pere Mata, Reus, Spain; 10 Neuriociències i Salut Mental, Institut d’Investigació Sanitària Pere Virgili-CERCA, Reus, Spain; 11 Department Medicina i Cirurgia, Universitat Rovira i Virgili, Tarragona, Spain; 12 Facultad de Medicina, Universidad Complutense de Madrid (UCM), Spain; 13 Department of Psychiatry, Instituto de Investigación Sanitaria Hospital, Madrid, Spain; 14 BIORABA, Department of Psychiatry, Hospital Universitario de Álava, CIBERSAM, UPV/EHU, Vitoria, Spain; 15 Research Group on Personal Autonomy, Dependency and Severe Mental Disorders, INCLIVA Biomedical Research Institute, Valencia, Spain; 16 Teaching Unit of Psychiatry and Psychological Medicine, Department of Medicine, University of Valencia, Valencia, Spain; 17 Department of Developmental and Educational Psychology, Faculty of Psychology and Speech and Language Therapy, University of Valencia, Valencia, Spain; 18 Research Unit, Parc Sanitari Sant Joan de Déu, Sant Boi de Llobregat, Spain; 19 MERITT Research Group, Institut de Recerca Sant Joan de Déu, Esplugues de Llobregat, Spain; 20 Department of Psychology, University of Oviedo, Oviedo, Spain; 21 Department of Psychiatry, Servicio de Salud del Principado de Asturias (SESPA), Oviedo, Spain; 22 Psychiatric Research Group, Instituto de Investigación Sanitaria del Principado de Asturias (ISPA), Oviedo, Spain; 23 Behavioral and Mental Disorders Neuroscience Unit, INEUROPA, Oviedo, Spain; 24 Department of Neuroscience, University of the Basque Country (UPV/EHU), Leioa, Spain; 25 Department of Psychiatry, Basurto University Hospital, OSI Bilbao-Basurto, Bilbao, Spain; 26 Early Psychosis: Intervention and Clinical-detection (EPIC) Lab, Department of Psychosis Studies, King’s College London, London, UK; 27 Psychiatry Service, Biobizkaia Health Research Institute, Barakaldo, Spain; 28 Department of Child and Adolescent Psychiatry, Institute of Psychiatry, Psychology & Neuroscience (IoPPN), King’s College London, London, UK; 29 Hospitalarias Arturo Soria Foundation, Madrid (Spain); 30 Institut de Salut Mental, Hospital del Mar, Barcelona, Spain; 31 Mental Health Research Group, Hospital del Mar Research Institute, CIBERSAM, Barcelona, Spain; 32 Department of Psychiatry, Zucker School of Medicine at Hofstra/Northwell, Hempstead, NY, USA; 33 Department of Psychiatry, Mental Health and Addictions, Hospital Universitari Santa Maria, Lleida, Spain; 34 Biological Foundations of Mental Disorders Group, Institut de Recerca Biomèdica (IRB) de Lleida, Lleida, Spain; 35 Department of Psychiatry, Hospital Álvaro Cunqueiro, Servicio Galego de Saúde (SERGAS), Vigo, Spain; 36 Translational Neuroscience Group, Galicia Sur Health Research Institute (IIS Galicia Sur), SERGAS-UVIGO, Vigo, Spain; 37 Department of Psychiatry, HGU Gregorio Marañón, Madrid, Spain; 38 Department of Psychiatry, Hospital Dr. Rodríguez Lafora, Madrid, Spain; 39 Department of Psychiatry, Universidad Francisco de Vitoria. Madrid, Spain; 40 Mental Health Group, Consorci Corporació Sanitària Parc Taulí – I3PT – CIBERSAM. Sabadell, Barcelona, Spain; 41 Departament de Psiquiatria i Medicina Legal, Universitat Autònoma de Barcelona, Barcelona, Spain; 42 Department of Psychiatry, University Hospital Virgen del Rocio, Andalusian Health Service, Seville, Spain

**Keywords:** BAC app, cognitive assessment, first-episode psychosis, normative data, schizophrenia, validation study

## Abstract

**Background:**

Cognitive impairment is central to psychosis and strongly linked to functional outcomes. The Brief Assessment of Cognition (BAC) app is a tablet-based, automated tool for assessing key cognitive domains but has not been validated in Spanish-speaking populations or across illness stages.

**Methods:**

A total of 402 participants (117 with first-episode psychosis [FEP], 125 with schizophrenia, and 160 controls) completed the BAC app along with clinical and functional assessments. We evaluated internal consistency, group differences, convergent and discriminant validity, and the effects of sex, age, and education. Normative percentiles were derived from controls.

**Results:**

The BAC app showed good internal consistency across groups (α = 0.76–0.87) and effectively differentiated individuals with psychosis from controls (area under the curve [AUC] = 0.862), with performance declining from controls to FEP to schizophrenia. Discrimination between FEP and schizophrenia was limited (AUC = 0.649). BAC App correlated positively with estimated intelligence quotient and functional capacity, and negatively with symptom severity, particularly in FEP. Performance varied by age, sex, and education, supporting the need for stratified normative data.

**Conclusions:**

The BAC app showed strong reliability and validity for cognitive assessment in Spanish-speaking individuals with psychosis. Its brevity, automated scoring, and normative data support its clinical and research applications for cognitive screening, monitoring, and treatment evaluation.

## Introduction

Cognitive impairment is a core feature of psychotic disorders, present from the early stages of illness and often persisting throughout its course [1]. Longitudinal studies have shown that deficits in cognitive domains such as attention, memory, and executive functioning predict a broad range of functional outcomes in schizophrenia, including social functioning, occupational adjustment, independent living, self-care, quality of life, and overall community integration [2]. Consequently, accurate and efficient assessment of cognitive functioning is essential for early identification of difficulties, individualized treatment planning, and monitoring of therapeutic interventions [3].

Numerous instruments are currently used to assess cognition in individuals with psychotic disorders, including standardized cognitive assessment batteries such as the MATRICS Consensus Cognitive Battery, which is considered the gold standard for evaluating cognitive impairment in schizophrenia [4]. While these tools are well-validated and widely used in research, they present notable limitations in clinical practice due to their administration time and need for staff time and training. To address these barriers, the Brief Assessment of Cognition in Schizophrenia (BACS) was developed as a rapid, reliable, and portable alternative that focuses on cognitive domains most impaired and most strongly related to functional outcomes in schizophrenia, including verbal memory, working memory, attention, executive functions, and motor speed [5]. Requiring approximately 30 minutes to complete, the BACS has demonstrated high test–retest reliability, minimal practice effects, and strong concurrent validity with standard batteries.

The growing availability of digital tools has fostered the development of assessments that offer automated scoring, standardized instruction delivery, and reduced rater-related variability, making them particularly useful in both research and clinical contexts. Several brief computerized neurocognitive test batteries have been validated in individuals with psychotic disorders [6–8]. Within this context, the Brief Assessment of Cognition (BAC) app was developed as a fully digital adaptation of the BACS, aiming to retain the original instrument’s psychometric strengths while taking advantage of the practical benefits of tablet-based administration. The BAC app includes digital versions of the six BACS subtests assessing verbal memory (list learning and recall), working memory (digit sequencing), motor speed (token motor task), verbal fluency (semantic and phonemic), attention and processing speed (symbol coding), and executive functioning (Tower of London). Each task is self-administered on a tablet device and features standardized audio and visual instructions, automated timing, and immediate scoring, reducing examiner bias and enhancing feasibility in routine settings. This format minimizes the need for trained examiners, eliminates rater-related variability, and supports consistent administration across settings. Its validation study has shown high concordance with the paper version, preserved sensitivity to cognitive impairment, and maintained strong associations with functional outcomes [9].

Beyond its psychometric properties, the BAC app exemplifies a broader movement toward the modernization of cognitive assessment in schizophrenia and related disorders. This transition reflects a growing interest in tools that combine psychometric rigor with practical benefits such as remote administration, ecological validity, and reduced burden on clinicians and patients [10–12]. Recent studies have demonstrated the feasibility and utility of mobile-based cognitive tools to monitor functioning longitudinally and across diverse settings, enhancing accessibility and enabling scalable, cross-cultural applications [13, 14]. The European Psychiatric Association emphasizes the necessity of such innovation in guiding more accurate and comprehensive assessments, suggesting that these digital adaptations can greatly improve both research and clinical practice by integrating real-world cognitive assessments [11]. In this context, the BAC App, represents a practical and innovative solution to the logistical barriers of traditional assessment approaches.

However, available evidence of the psychometric properties of the BAC app is limited to a single study conducted in a small clinical sample and did not differentiate between distinct stages of illness, such as first-episode psychosis (FEP) and chronic schizophrenia, limiting generalizability of its findings. Moreover, no normative data are currently available for Spanish-speaking populations nor has the influence of key demographic factors such as age, sex, and education been examined. Notably, the original validation did not assess internal consistency, leaving the reliability of the total composite score unverified—a crucial aspect for its use in clinical interpretation and longitudinal monitoring.

Thus, the present study aimed to provide a comprehensive validation of the BAC app in a Spanish-speaking sample that includes individuals with FEP, chronic schizophrenia, and healthy controls (HCs). Specifically, we seek to (i) examine the internal consistency of the BAC app; (ii) assess discriminative validity by comparing mean performance across groups (HCs, FEP, and schizophrenia) and evaluating the classification accuracy of the BAC app through receiver operating characteristic (ROC) curve analyses; (iii) evaluate convergent validity by examining correlations between BAC app scores and estimated intelligence quotient (IQ) and a functional capacity measure, and test the independence of BAC app scores from symptom severity; (iv) analyze the influence of key demographic variables (sex, age, and education) on BAC app performance within each group; and(v) subsequently develop stratified normative data for each BAC app subtest and the composite score based on the observed effects of these demographic variables.

## Methods

### Participants and procedure

Participants were recruited as part of a multicenter study in 15 public mental health centers and university hospitals across Spain to validate digital tools for assessing cognitive and functional capacity in individuals with psychotic disorders. The final sample included individuals diagnosed with chronic schizophrenia or FEP, identified through clinician referral based on convenience sampling, as well as HCs recruited to be comparable in age, sex, and educational level. A total of 403 participants were initially recruited, including 117 individuals with FEP, 125 with chronic schizophrenia, and 161 HCs. After excluding one control with missing BAC app data, the final sample comprised 402 individuals.

Participants were eligible if they: (1) were aged 18–60 years; (2) met diagnostic criteria for a schizophrenia spectrum disorder according to the Diagnostic and Statistical Manual of Mental Disorders, Fifth Edition (DSM-5); (3) had adequate Spanish proficiency; and (4) could provide informed consent. Exclusion criteria included (1) organic brain pathology or neurological illness, (2) intellectual disability (DSM-5), and (3) current or recent (past 6 months) substance dependence, assessed via the Comprehensive Assessment of Symptoms and History (CASH [15]). Participants were classified as FEP if they had initiated antipsychotic treatment within the past 3 years, a widely accepted threshold based on the “critical period” hypothesis, which posits that the first 3 years after illness onset represent a pivotal window for intervention [16, 17]; otherwise, they were classified as having chronic schizophrenia.

HCs met the same age and language criteria and were required to provide informed consent. Exclusion criteria included any current or past mental or neurological disorder, organic brain pathology, intellectual disability, substance use disorder, or psychotropic medication use.

Data were collected between July 2022 and December 2024. Participants with psychosis were recruited from routine clinical care settings across 15 public university-affiliated hospitals and mental health centers in Spain. These included early intervention programs for FEP, day hospitals, outpatient clinics, and acute inpatient units. Although recruitment was based on convenience sampling, the diversity of participating public sector services reflects typical clinical contexts of psychosis care in Spain. HCs were also recruited using convenience sampling strategies, including outreach to university students, hospital staff, and acquaintances of patients or clinicians. The absence of mental or neurological disorders was operationally defined as having no current or past mental disorders (including mood, anxiety, or psychotic disorders), no history of neurological illness, no substance use disorder (past or present), and no use of psychotropic medication. These criteria were confirmed through the CASH structured interview and participant self-report.

Trained research staff administered the assessments in a single session following standardized instructions. Diagnoses were established according to DSM-5 criteria through semistructured face-to-face clinical interviews conducted by trained clinicians at each site. Although no formal blinding was used, the BAC app was generally administered by a different team member than the one responsible for diagnosis. Written informed consent was obtained, and the study received ethical approval from the ethics committee of the principal center (code: PI20/00066) and from the corresponding ethics committees at each participating site.

### Measures

#### BAC app

The BAC app is a tablet-based version of the BACS [5, 9], designed to assess six cognitive domains relevant to clinical populations: episodic memory, working memory, verbal fluency, processing speed, executive functioning, and psychomotor speed. The full battery required approximately 30 minutes to complete. Raw scores from each subtest were converted into T-scores based on the HC group distribution. These T-scores were then averaged to generate a cognitive composite score reflecting overall performance. Higher scores indicate better cognitive performance across all subtests.

#### Virtual Reality Functional Capacity Assessment Tool (VRFCAT)

The VRFCAT is a computerized performance-based measure developed to assess functional capacity through the simulation of everyday tasks in a realistic virtual environment [18]. A Spanish-translated version of the VRFCAT was administered on a tablet. In the present study, only the total time to completion was used for analysis, with higher times indicating poorer functional capacity. Internal consistency of this composite score was acceptable to good across groups: for the total sample, ω = 0.80; for the clinical group, ω = 0.76; and for controls, ω = 0.71.

#### Positive and Negative Syndrome Scale (PANNS)

In the present study, the validated Spanish version of the PANSS was used [19, 20]. The PANSS includes three subscales: positive symptoms (seven items), negative symptoms (seven items), and general psychopathology (16 items).

#### *Vocabulary subtest of the Wechsler Adult Intelligence Scale – Third* Edition *(WAIS-III)*

The vocabulary subtest from the Spanish version of the WAIS-III was used as an estimate of premorbid IQ [21]. Participants are required to define a series of words that increase in difficulty, providing a measure of verbal knowledge and crystallized intelligence. Raw scores were converted to age-adjusted scaled scores using normative data from the WAIS-III manual.

### Sociodemographic and clinical variables

Sociodemographic data were collected, including age, sex, ethnicity, educational level, and current employment status. Clinical status was obtained through a structured interview that included mental diagnosis based on DSM-5 criteria, age of onset, duration of untreated illness (DUI), and duration of untreated psychosis (DUP).

### Data analysis

Descriptive statistics were computed by group (FEP, schizophrenia, and HCs), including means with standard deviations, medians, interquartile ranges, skewness, and kurtosis. Group comparisons used χ^2^ for categorical and *t*-tests, analyses of variance (ANOVAs), or nonparametric tests (Mann–Whitney U, Kruskal–Wallis) for continuous variables.

Raw scores on each BAC app subtest were converted into T-scores (mean = 50, SD = 10) using the HC sample as a normative reference group. This standardization facilitated the computation of a composite cognitive score, calculated as the average of the six subtest T-scores.

Internal consistency of the BAC app was estimated using Cronbach’s alpha for each group and the total sample. Intercorrelations between subtests were also examined to assess construct coherence.

To evaluate group differences in cognitive performance, one-way ANOVAs were conducted on T-scores and the composite score. Post hoc comparisons were conducted using Tukey’s HSD test when the assumption of homogeneity of variances (Levene’s test, *p* > 0.05) was met and the Games–Howell test when it was not (*p* < 0.05). ROC curve analyses were performed to assess the discriminative validity of BAC app scores in distinguishing between the clinical and the control groups, as well as between FEP and schizophrenia subgroups. Area under the curve (AUC), sensitivity, specificity, and optimal cutoff points (Youden Index) were reported.

To develop normative data, we first examined the effects of sex, age, and years of education on BAC app performance within each group. Based on the observed associations, quantile regression analyses were conducted on the HC sample to generate normative percentile scores (10th, 25th, 50th, 75th, and 90th) for the six BAC app subtests. Percentiles were stratified by sex, age group, and educational level. Age and education were categorized into three levels each to ensure clinically meaningful distinctions and adequate sample sizes.

Finally, we analyzed the relationships between estimated IQ, functional capacity (VRFCAT total time to completion), and clinical symptoms, assessed through the positive, negative, and general subscales of the PANSS. All statistical tests were two-tailed, with the significance threshold set at *p* < 0.05. Analyses were conducted using IBM SPSS Statistics (version 26).

## Results

### Sociodemographic and clinical characteristics of the sample

Table 1 presents the demographic and clinical characteristics of participants in the FEP, schizophrenia, and HC groups. Significant differences were found across groups in age, years of education, ethnicity, marital status, and employment status. In contrast, sex distribution did not differ significantly between groups.

**Table 1.** Demographic and clinical characteristics of FEP, schizophrenia, and healthy controls

	FEP (*n* = 117)	Schizophrenia (*n* = 125)	HC (*n* = 160)	Statistic	*p*-Value
Age, Md (IQR)	24.5 (20–33)	40.0 (31–48)	31.0 (26–38.5)	H = 67.521	<0.001
Sex, *n* (%)				χ^2^ = 5.014	0.082
Male	74 (63.2)	85 (68.0)	88 (55.3)		
Female	43 (36.8)	40 (32.0)	72 (44.7)		
Years of education, Md (IQR)	14.0 (11–17)	12.0 (10–16)	15.5 (12–19)	H = 23.219	<0.001
Marital status, *n* (%)				χ^2^ = 23.628	<0.001
Single	94 (81.0)	101 (81.5)	95 (60.9)		
Married	15 (12.9)	14 (11.3)	49 (31.4)		
Other	7 (6.0)	9 (7.3)	12 (7.7)		
Ethnicity, *n* (%)				χ^2^ = 30.598	<0.001
Caucasian	64 (54.7)	100 (80.0)	131 (81.9)		
Latino	48 (41.0)	21 (16.8)	27 (16.9)		
Other	5 (4.3)	4 (3.2)	3 (1.7)		
Employment status, *n* (%)				χ^2^ = 172.941	<0.001
Student	33 (28.7)	13 (10.5)	27 (16.9)		
Employed	41 (35.7)	23 (18.5)	117 (73.1)		
Unemployed	24 (20.9)	20 (16.1)	9 (5.6)		
Disability	4 (3.5)	50 (40.3)	2 (1.3)		
Other	13 (11.3)	18 (14.5)	5 (3.1)		
DUP, Md (IQR)	2.0 (1–5.5)	2.0 (1–24)	—	U = 5993.5	0.031
DUI, Md (IQR)	6.0 (2–24)	12.0 (1–72)	—	U = 6279.0	0.193
PANSS positive, Md (IQR)	11.0 (7–17)	11.0 (8–15)	—	U = 6703.5	0.981
PANSS negative, Md (IQR)	12.0 (7–18)	15.0 (10–20)	—	U = 5404.5	0.038
PANSS general, Md (IQR)	27.0 (20–33.8)	25.0 (20–31.8)	—	U = 6121.0	0.469

Abbreviations: DUI, duration of untreated illness (in months); DUP, duration of untreated psychosis (in months); FEP, first-episode psychosis; H, Kruskal–Wallis test statistic; HC,= healthy controls; IQR, interquartile range (25th–75th percentile); Md, median; PANSS, Positive and Negative Syndrome Scale; U, Mann–Whitney U test statistic.

Descriptive statistics for each BAC app subtest and the composite score are presented in Table 2 for each group. Distributions across variables were generally symmetric, although some subtests (e.g., tower of London) showed notable skewness and kurtosis, particularly in the control group.

**Table 2.** Descriptive statistics for BAC app scores in FEP, schizophrenia, and healthy control groups

BAC app	Mean	SD	Median	IQR	Skewness	Kurtosis
FEP						
Verbal memory	39.8	11.4	41.0	33.0–48.0	−0.5	0.4
Digit sequencing	17.3	4.9	17.0	13.0–21.0	0.3	−0.8
Token motor	93.7	32.6	90.0	69.0–118.0	0.2	−0.4
Verbal fluency	42.3	13.2	43.0	34.0–50.0	0.0	0.7
Symbol coding	42.5	12.3	44.0	34.0–50.5	−0.2	0.1
Tower of London	16.1	4.1	18.0	14.0–19.0	−1.4	2.3
Composite score	40.6	8.4	41.1	35.3–45.4	−0.4	0.4
Schizophrenia						
Verbal memory	33.3	13.1	33.0	24.5–42.0	−0.1	−0.3
Digit sequencing	16.3	5.4	16.0	13.0–20.0	−0.2	0.2
Token motor	68.5	33.9	64.0	42.0–90.0	0.3	−0.6
Verbal fluency	40.0	12.9	39.0	32.0–48.0	0.2	1.0
Symbol coding	36.4	12.8	37.0	28.0–46.5	−0.3	−0.1
Tower of London	14.6	5.3	16.0	12.0–18.0	−1.1	0.8
Composite score	35.8	9.3	36.8	30.4–41.8	−0.7	1.1
Healthy controls						
Verbal memory	48.9	10.1	49.5	42.0–56.8	−0.3	−0.1
Digit sequencing	21.9	4.5	23.0	19.0–25.0	−0.9	0.2
Token motor	118.6	32.0	121.0	98.0–141.5	−0.2	0.3
Verbal fluency	55.9	13.8	56.0	47.0–65.0	0.1	0.9
Symbol coding	55.0	12.5	55.0	48.0–62.0	0.3	1.2
Tower of London	18.7	2.7	19.0	18.0–21.0	−1.9	7.4
Composite score	50.0	6.8	51.0	46.4–53.8	−0.8	1.7

Abbreviations: BAC app, Brief Assessment of Cognition app; FEP, first-episode psychosis; IQR, interquartile range (25th–75th percentile); SD, standard deviation.

*Note*: Subtest scores are raw scores. The composite score is the average of T-scores from the six BAC App domains, standardized using healthy control group data.

### Internal consistency

Internal consistency was acceptable to good across groups. For the FEP group, Cronbach’s α = 0.83 (95% confidence interval [CI] [0.77–0.87]); for the schizophrenia group, α = 0.81 (95% CI [0.73–0.86]); for the control group, α = 0.76 (95% CI [0.68–0.82]); and for the total sample, α = 0.87 (95% CI [0.85–0.89]).

Additionally, all BAC app subtests showed statistically significant positive correlations across the three groups (FEP, schizophrenia, and controls), with the strongest associations observed between each subtest and the global composite score (*r* = 0.63–0.82). Intersubtest correlations were generally moderate to high (*r* = 0.28–0.64).

### Group differences in BAC app performance

As shown in Table 3, there were statistically significant group effects for all subtests and the composite score (all *p* < 0.001). Post hoc comparisons revealed that HCs outperformed both clinical groups across all domains, whereas participants with FEP showed significantly better performance than those with schizophrenia on most subtests, a pattern that was especially evident in the composite score.

**Table 3.** ANOVA results and post hoc comparisons of BAC app subtests and composite score across FEP, SCZ, and HC groups

Test	FEP, mean (SD)	SCZ, mean (SD)	HC, mean (SD)	*F*(2, 399)	*p*-Value	Post hoc^a^
Verbal memory	39.80 (11.39)	33.28 (13.06)	48.85 (10.10)	66.33	<0.001	HC > FEP*** HC > SCZ*** FEP > SCZ***
Digit sequencing	17.28 (4.88)	16.34 (5.37)	21.85 (4.48)	52.63	<0.001	HC > FEP*** HC > SCZ***
Token motor	93.66 (32.56)	68.48 (33.90)	118.59 (32.00)	82.40	<0.0001	HC > FEP*** HC > SCZ*** FEP > SCZ***
Verbal fluency	42.26 (13.16)	39.96 (12.94)	55.93 (13.75)	60.68	<0.001	HC > FEP*** HC > SCZ***
Symbol coding	42.51 (12.33)	36.38 (12.81)	54.99 (12.47)	82.24	<0.001	HC > FEP*** HC > SCZ*** FEP > SCZ**
Tower of London	16.10 (4.14)	14.63 (5.33)	18.71 (2.67)	36.63	<0.001	HC > FEP*** HC > SCZ*** FEP > SCZ*
Composite score	40.56 (8.36)	35.80 (9.27)	50.00 (6.75)	114.99	<0.001	HC > FEP*** HC > SCZ*** FEP > SCZ***

Abbreviations: FEP first-episode psychosis; SCZ, schizophrenia; HC, healthy controls; SD, standard deviation.

*Note*: Subtest scores are raw scores. The CS is the average of T-scores from the six BAC app domains, standardized using healthy control group data.

a Post hoc comparisons were conducted using Tukey’s HSD test when the assumption of homogeneity of variances (Levene’s test, *p* > 0.05) was met, and Games–Howell test when it was not (*p* < 0.05).

* *p* < 0.05.

** *p* < 0.01.

*** *p* < 0.001.

### ROC curve analyses

**Figure 1.**
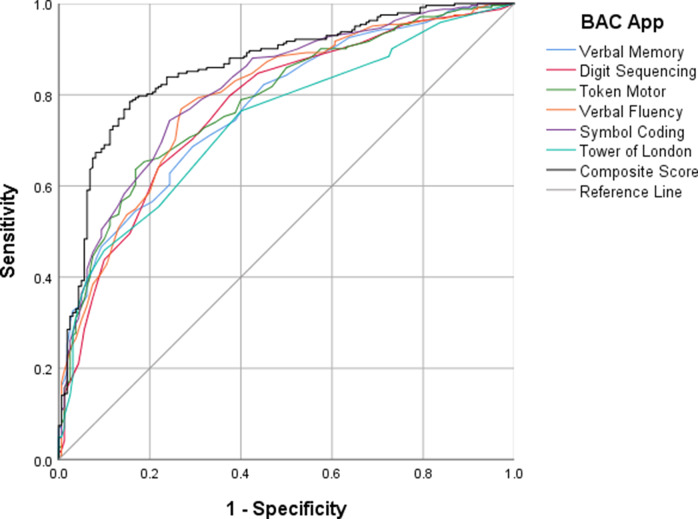
Receiver operating characteristic (ROC) curves for Brief Assessment of Cognition (BAC) app subtests and composite score: clinical (first-episode psychosis [FEP] + schizophrenia) group versus controls. ROC curves comparing individuals with psychosis (FEP and schizophrenia combined) to healthy controls on BAC app subtest T-scores and the composite score. The composite score showed the highest discriminative validity (area under the curve [AUC] = 0.862), followed by symbol coding (AUC = 0.814), verbal fluency (AUC = 0.794), token motor (AUC = 0.785), verbal memory (AUC = 0.774), digit sequencing (AUC = 0.772), and tower of London (AUC = 0.741). The diagonal reference line (AUC = 0.50) represents chance-level performance.

ROC curve analyses were conducted to evaluate the discriminative validity of the BAC app subtest T-scores and composite score. As shown in Figure 1, when comparing the clinical group (individuals with FEP and schizophrenia combined) to HCs, the composite score showed good classification accuracy, yielding an AUC of 0.862 (95% CI [0.826–0.899]). The optimal cutoff point of 47, identified using the Youden index (J = 0.63), resulted in a sensitivity of 77.3% and a specificity of 85.0%. Among the individual subtests, symbol coding demonstrated the highest discriminative power (AUC = 0.814).

**Figure 2.**
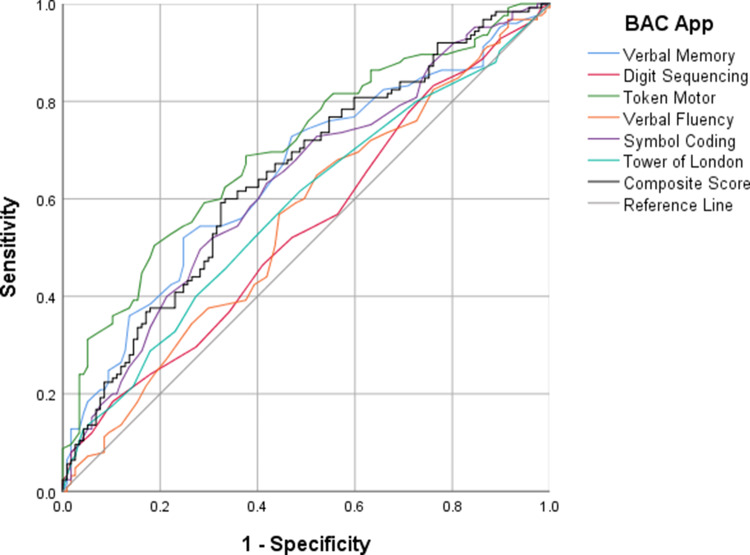
Receiver operating characteristic (ROC) curves for Brief Assessment of Cognition (BAC) app subtests and composite score: first-episode psychosis (FEP) versus schizophrenia. *Note.* ROC curves comparing individuals with FEP to those with schizophrenia on BAC app subtest T-scores and the composite score. The composite score showed poor discriminative validity (area under the curve [AUC] = 0.649), with the token motor subtest yielding the highest AUC (0.702) followed by verbal memory (AUC = 0.650), symbol coding (AUC = 0.632), tower of London (AUC = 0.580), verbal fluency (AUC = 0.553), and digit sequencing (AUC = 0.541). The diagonal reference line (AUC = 0.50) represents chance-level performance.

To examine the ability of the BAC app to distinguish between individuals with FEP and those with schizophrenia, a second set of ROC analyses was performed within the clinical sample (see Figure 2). The composite score showed poor discriminative ability (AUC = 0.649, 95% CI [0.580–0.718]), with an optimal cutoff of 45 (Youden index = 0.30), yielding a sensitivity of 68.6% and a specificity of 61.6%. The token motor subtest provided the highest AUC in this comparison (0.702), while the remaining subtests ranged from 0.541 to 0.650, indicating limited accuracy in differentiating between these clinical subgroups.

### Associations with functional capacity performance, IQ, and clinical symptoms

As shown in Table 4, BAC app scores were significantly associated with both estimated IQ and functional capacity across all groups, with stronger correlations generally observed in clinical groups, indicating that better cognitive performance was associated with higher IQ and better functional capacity.

**Table 4.** Correlations between BAC app and estimated IQ/VRFCAT

	Estimated IQ	VRFCAT time	PANSS positive	PANSS negative	PANSS general
First-episode psychosis					
Verbal memory	0.40***	−0.352***	−0.19*	−0.21*	−0.19*
Digit sequencing	0.31**	−0.127	−0.28**	−0.29**	−0.29**
Token motor	0.21*	−0.426***	−0.07	−0.11	−0.07
Fluency	0.28**	−0.065	−0.17	−0.39***	−0.30**
Symbol coding	0.22*	−0.368***	−0.14	−0.10	−0.12
Tower of London	0.18	−0.218*	−0.21*	−0.14	−0.21*
Composite score	0.38***	−0.359***	−0.27**	−0.29**	−0.30**
Schizophrenia					
Verbal memory	0.48***	−0.387***	−0.09	−0.17	−0.06
Digit sequencing	0.51***	−0.310**	−0.11	−0.20*	−0.12
Token motor	0.15	−0.498***	−0.11	−0.09	0.01
Fluency	0.46***	−0.247**	−0.22*	−0.32***	−0.28**
Symbol coding	0.32**	−0.622***	−0.12	−0.16	−0.13
Tower of London	0.32**	−0.572***	−0.01	−0.15	−0.14
Composite score	0.49***	−0.614***	−0.11	−0.21*	−0.15
Health controls					
Verbal memory	0.27**	−0.172*			
Digit sequencing	0.30***	−0.185*			
Token motor	0.10	−0.225**			
Fluency	0.25**	−0.214**			
Symbol coding	0.23**	−0.301***			
Tower of London	0.05	−0.110			
Composite score	0.31***	−0.291***			

Abbreviations: BAC app, Brief Assessment of Cognition app; IQ, estimated intelligence quotient (vocabulary subtest); VRFCAT, Virtual Reality Functional Capacity Assessment Tool.

*Note*: Spearman’s rho correlations. * *p* < 0.05, ** *p* < 0.01, *** *p* < 0.001. Higher VRFCAT Time indicates poorer functional capacity.

Regarding clinical symptoms, negative associations were observed between BAC app performance and all three PANSS dimensions, with correlation magnitudes ranging from small to moderate. While not all correlations reached statistical significance, those that did suggest that greater symptom severity is linked to poorer cognitive functioning. Overall, associations tended to be stronger in the FEP group than in the schizophrenia group.

### Normative performance on the BAC app

As a preliminary step to determine whether stratification of normative data was warranted, we examined the effects of sex, age, and education on BAC app performance using the composite score as the primary outcome. For this type of analysis, we opted to use the composite score rather than analyzing the six subtests separately, as it provides a reliable and sensitive global indicator of cognitive performance and minimizes multiple testing issues. In the FEP group, women obtained significantly higher composite scores than men (*t*(106.0) = −2.36, *p* = 0.020), whereas no significant sex differences were found in the schizophrenia or control groups. Age was negatively correlated with the composite score in both the schizophrenia (ρ = −0.30, *p* = 0.001) and control (ρ = −0.24, *p* = 0.003) groups. Years of education were positively associated with the composite score across all groups: FEP (ρ = 0.34, *p* < 0.001), schizophrenia (ρ = 0.41, *p* < 0.001), and controls (ρ = 0.40, *p* < 0.001). These findings support the need to stratify normative data according to these key demographic variables. Table 5 shows normative percentile scores (10th, 25th, 50th, 75th, and 90th) derived from the HC sample and stratified by sex, age group, and years of education.

**Table 5.** Normative percentile scores for the BAC app subtests and CS, stratified by sex, age group, and education level

			Women							Men						
Age group (years)	Education (years)	Percentile	VM	DS	TM	VF	SC	TL	CS	VM	DS	TM	VF	SC	TL	CS
18–29	5–9	10	35	11	40	36.0	46	11	30.8	31	10	42	39.0	42	11	29.8
		25	36	17	76	33.0	46	12	34.1	33	18	74	38.0	43	12	35.2
		50	41	18	100	48.0	51	18	44.3	39	19	102	52.0	49	18	46.0
		75	51	24	136	59.0	59	20	50.9	53	25	142	63.0	56	21	52.8
		90	59	27	150	68.0	60	18	53.7	61	28	158	72.0	57	19	54.4
	10–14	10	40	14	72	38.0	45	17	44.1	36	13	74	39.5	41	17	43.0
		25	40	18	104	41.0	53	18	45.7	37	19	102	45.0	50	18	46.8
		50	47	21	130	50.0	58	19	48.8	45	22	132	53.0	57	19	50.5
		75	53	24	146	57.0	67	21	51.5	55	25	152	60.0	66	22	53.5
		90	61	26	168	64.0	73	21	57.2	63	26	176	66.0	77	22	57.9
	15+	10	43	16	84	38.5	49	18	44.4	39	15	86	42.0	45	18	43.4
		25	47	19	112	47.0	53	19	49.5	44	22	110	53.0	50	19	50.5
		50	53	22	128	54.0	59	19	52.6	51	24	130	62.0	58	19	54.3
		75	58	25	152	63.0	69	21	56.6	60	26	158	69.0	67	22	58.6
		90	65	27	172	74.0	75	21	59.3	67	27	180	78.0	78	22	60.0
30–44	5–9	10	33	11	40	36.0	46	11	31.0	29	10	42	39.0	42	11	29.9
		25	35	17	76	33.0	46	12	33.6	31	18	74	38.0	43	12	34.7
		50	39	18	100	48.0	51	18	43.3	37	19	102	52.0	49	18	45.0
		75	49	24	136	59.0	59	20	49.1	51	25	142	63.0	56	21	51.1
		90	57	27	150	68.0	60	18	51.4	59	28	158	72.0	57	19	52.1
	10–14	10	38	14	72	38.0	45	17	44.2	34	13	74	39.5	41	17	43.1
		25	39	18	104	41.0	53	18	45.2	35	19	102	45.0	50	18	46.3
		50	45	21	130	50.0	58	19	47.8	43	22	132	53.0	57	19	49.5
		75	51	24	146	57.0	67	21	49.8	53	25	152	60.0	66	22	51.7
		90	59	26	168	64.0	73	21	54.9	61	26	176	66.0	77	22	55.6
	15+	10	41	16	84	38.5	49	18	44.6	37	15	86	42.0	45	18	43.5
		25	47	19	112	47.0	53	19	49.0	45	22	110	53.0	50	19	50.0
		50	53	22	128	54.0	59	19	51.6	52	24	130	62.0	58	19	53.3
		75	58	25	152	63.0	69	21	54.9	59	26	158	69.0	67	22	56.8
		90	65	27	172	74.0	75	21	57.0	62	27	180	78.0	78	22	57.7
45+	5–9	10	30	11	40	36.0	46	11	28.2	26	10	42	39.0	42	11	27.2
		25	31	17	76	33.0	46	12	29.3	28	18	74	38.0	43	12	30.4
		50	35	18	100	48.0	51	18	39.8	31	19	102	52.0	49	18	41.5
		75	45	24	136	59.0	59	20	44.5	47	25	142	63.0	56	21	46.4
		90	53	27	150	68.0	60	18	48.8	55	28	158	72.0	57	19	49.5
	10–14	10	35	14	72	38.0	45	17	41.5	31	13	74	39.5	41	17	40.4
		25	36	18	104	41.0	53	18	40.9	32	19	102	45.0	50	18	42.0
		50	41	21	130	50.0	58	19	44.3	39	22	132	53.0	57	19	46.0
		75	51	24	146	57.0	67	21	45.1	49	25	152	60.0	66	22	47.0
		90	59	26	168	64.0	73	21	52.3	57	26	176	66.0	77	22	53.0
	15+	10	38	16	84	38.5	49	18	41.9	34	15	86	42.0	45	18	40.8
		25	44	19	112	47.0	53	19	44.7	40	22	110	53.0	50	19	45.7
		50	49	22	128	54.0	59	19	48.1	43	24	130	62.0	58	19	49.8
		75	54	25	152	63.0	69	21	50.2	56	26	158	69.0	67	22	52.1
		90	61	27	172	74.0	75	21	54.4	60	27	180	78.0	78	22	55.1

Abbreviations: CS, composite score; DS, digit sequencing; SC, symbol coding; TL, tower of London; TM, token motor; VF, verbal fluency; VM, verbal memory.

*Note*: Subtest scores are raw scores. The CS is the average of T-scores from the six BAC app domains, standardized using healthy control group data. Percentile scores were calculated independently using quantile regression models. Minor reversals in percentile order (e.g., P90 < P75) were observed only in the tower of London domain and do not indicate data errors. Clinicians are advised to interpret these cases cautiously and consider the overall pattern of scores rather than isolated percentile differences in this domain.

## Discussion

This study aimed to validate the BAC app in a Spanish-speaking sample, including individuals with FEP, chronic schizophrenia, and HCs. The BAC app showed good discriminative validity in distinguishing individuals with psychosis from HCs, showing a clear gradient of impairment from controls to FEP to individuals with chronic schizophrenia. Internal consistency of the composite score was acceptable to good across all groups, supporting its use as a global index of cognitive performance. The BAC app showed small-to-moderate positive associations with higher IQ and better functional capacity, and negative associations with greater symptom severity. In addition, because performance on the BAC app varied significantly by sex, age, and education, normative data were stratified accordingly to support meaningful interpretation in clinical settings.

These findings are broadly consistent with the initial validation study of the BAC app by Atkins et al. [9], which reported strong concordance with the paper-based BACS and significant associations with functional outcomes. Individuals with FEP performed better than those with chronic schizophrenia across most BAC app subtests, except digit sequencing and verbal fluency. While this cross-sectional gradient might suggest cumulative cognitive deterioration, longitudinal studies have produced mixed findings regarding the course of cognitive functioning following illness onset [1, 2, 22–24]. Some short-term studies report gains likely due to practice effects [25]. Medium- to long-term studies show stability in most domains, except for declines in verbal memory, and mixed findings in executive functions [22]. Studies with follow-up periods beyond 10 years report modest deterioration in verbal and visuospatial memory, while processing speed and executive functions tend to remain stable [26–28]. Together with findings from data-driven subgroup analyses – such as latent class or growth mixture modeling – identifying stable, improving, or declining trajectories [29], this suggests that the differences observed in our study are more likely due to between-group variability (e.g., illness duration, treatment, and comorbidities) rather than uniform cognitive decline within individuals over time.

ROC curve analyses confirmed that the BAC app had good discriminative accuracy in differentiating individuals with psychosis from HCs (AUC = 0.862), with the composite score offering the highest classification performance. Among the individual subtests, symbol coding, a task that strongly depends on processing speed, yielded the highest AUC (0.814), suggesting that it may be particularly sensitive to illness-related cognitive impairment. This is in line with previous findings indicating that symbol coding is especially sensitive to cognitive deficits in psychotic disorders [30]. However, when attempting to distinguish between FEP and schizophrenia, the composite score showed only modest discriminative ability (AUC = 0.649), reflecting poor classification accuracy. As reported in prior literature, cognitive deficits are already evident in the early stages of psychosis [1] and, together with the heterogeneity of cognitive trajectories over time, may help to explain the modest discriminative power of the BAC app in separating early- from late-stage illness, despite statistically significant differences in group means.

When compared to other brief computerized batteries validated in psychosis, including the CogState Battery [8], the MyCognition Quotient [6], and PsyCog [7], the BAC app shows a pattern of results that overlaps in key areas, such as discriminative validity and sensitivity to cognitive deficits while differing in terms of the specific cognitive domains assessed, the mode of task presentation, and the rationale underlying their development. The convergence of psychometric properties across tools with different designs may reflect a shared capacity to detect broad cognitive impairment in psychosis, rather than the measurement of distinct cognitive processes. Consistent with previous findings suggesting a predominant general cognitive factor in psychosis [30], the high internal consistency and intersubtest correlations of the BAC App support its validity as a global measure of cognitive functioning. Although BAC app scores were only moderately correlated with estimated IQ, this is consistent with the fact that vocabulary-based estimates primarily reflect crystallized intelligence and may not fully represent the broader range of abilities encompassed by the general factor, such as processing speed, working memory, and executive function. In line with this interpretation, performance on a single task, such as symbol coding, has been found to explain a substantial proportion of global cognitive variance in FEP [30–32], reinforcing the idea that commonly used tasks may be more effective at detecting generalized impairment than at identifying distinct cognitive profiles [33, 34].

Regarding associations with clinical symptoms, in individuals with FEP, the BAC app composite score showed significant negative correlations with all three PANSS dimensions – positive, negative, and general – indicating that greater symptom severity across domains was associated with poorer cognitive performance. In contrast, among patients with chronic schizophrenia, only negative symptoms were significantly correlated with the composite score. This divergence suggests that the relationship between symptomatology and cognitive functioning may be more dynamic and multifaceted in the early stages of psychosis, whereas in chronic stages, cognitive impairments appear more stable and primarily linked to enduring negative symptoms. Importantly, the generally low magnitude of these correlations supports the discriminant validity of the BAC app, suggesting that it captures cognitive functioning beyond the influence of symptom severity.

Demographic variables such as sex, age, and education had an impact on BAC app performance across groups. The finding that women scored higher than men in FEP suggests that sex-related cognitive advantages, as previously described in psychosis [35], may be more evident in early stages of illness, but their impact may be outweighed by cumulative illness-related neurobiological burden, long-term antipsychotic treatment, and social disengagement, leading to a reduction in sex-related cognitive differences in chronic schizophrenia. The absence of sex differences among HCs may reflect ceiling effects in a cognitively intact population. Similarly, older age was associated with poorer performance across groups, with the most pronounced decline observed in the schizophrenia group, consistent with prior evidence of accelerated brain aging in psychosis [36]. Consistent with prior research, higher education was associated with better BAC app performance across groups, supporting its role as a cognitive reserve factor [37]. Given these influences, stratified norms by sex, age, and education are essential for accurate score interpretation.

Several limitations should be acknowledged. First, although the overall sample size was adequate for group comparisons, stratification of the normative data by sex, age, and education resulted in reduced cell sizes for some percentile estimations. Second, participants were recruited through convenience sampling, which may limit the generalizability of the findings. Although the control sample was carefully matched and screened to exclude individuals with mental, neurological, or substance-related disorders, participants were not selected through random population-based sampling procedures. This methodological limitation constrains the generalizability of the normative data to the broader Spanish population. Therefore, while the normative percentiles derived from this sample can be valuable for research- and group-level comparisons, they should be interpreted with caution when used for clinical purposes. Furthermore, given the limited evidence linking positive symptom severity to cognitive performance, and the practical limitations of assessing cognition during acute episodes, our findings primarily reflect cognitive functioning in nonacute stages of psychosis. Further studies are needed to evaluate the validity and reliability of the BAC app in highly symptomatic patients during acute phases, although it remains unclear whether cognitive performance under such conditions can meaningfully reflect a patient’s underlying cognitive functioning. Third, group comparisons revealed significant sociodemographic differences in age and educational attainment. While these differences represent a limitation, they are largely unavoidable in studies comparing individuals with FEP, chronic schizophrenia, and HCs. Fourth, convergent validity was assessed using the vocabulary subtest of the WAIS-III. Although vocabulary performance is an imperfect estimate of general cognitive ability, it remains a well-validated and stable proxy [38]. Finally, potential covariates such as medication use, age, and education were not controlled, as the aim was to capture real-world performance and provide ecologically valid normative data.

In conclusion, this study provides robust evidence supporting the reliability, validity, and clinical relevance of the BAC app as a brief, accessible digital tool for assessing cognitive functioning in Spanish-speaking populations with psychotic disorders, offering practical advantages for its use in clinical settings such as minimal staff training, automatic scoring, and standardized administration. Its ability to detect group differences, its associations with key clinical and functional outcomes, and the generation of stratified normative data all reinforce its utility for both research and applied practice. The BAC app remains a valuable instrument for screening cognitive impairment, tracking changes over time, and evaluating treatment response.

## Data Availability

The datasets generated and/or analyzed during the current study are available from the corresponding author upon reasonable request.
